# Heavy Drinking in Young Adulthood Correlates With Earlier Genetic Cardiomyopathy Diagnosis: Analyzing a Retrospective Survey

**DOI:** 10.1016/j.jacadv.2026.103222

**Published:** 2026-10-21

**Authors:** Julia Marine, Corine Sinnette, Alyx Vogle, Lauren Salbinski, Emily Johnson, Nicole K. Bart, Carolyn Y. Ho, Iswaree Balakrishnan, Charlotte Andersson, Neal K. Lakdawala

**Affiliations:** a Brigham and Women’s Hospital, Harvard Medical School, Boston, Massachusetts, USA; b St. Vincent’s Hospital, Sydney, Australia; c National Heart Centre Singapore, Singapore

**Keywords:** alcohol, genetic cardiomyopathy, penetrance

Alcohol consumption is pervasive in the United States. Heavy drinking, defined as consuming 5 or more drinks for men and 4 or more drinks for women within a 24-hour period, is particularly frequent among young adults. Even infrequent heavy drinking is associated with significant health risks, including cardiovascular events like atrial fibrillation, whereas chronic heavy alcohol use may lead to acquired alcoholic dilated cardiomyopathy (DCM).^1^ As a result, current clinical guidelines advise against heavy drinking (episodic or sustained) for all patients, especially those at an increased risk for cardiovascular events.^1^ However, the relationship between heavy drinking and age of onset of dilated or arrhythmogenic cardiomyopathy (DCM/ACM) in individuals who carry genetic variants that increase the risk for cardiomyopathy is not well understood. This study assesses the effect of heavy drinking in young adulthood (ages 21-30) on incident development of DCM/ACM in genetically predisposed individuals.

**What is the clinical question being addressed?**

What is the effect of heavy drinking in young adulthood on incident development of genetic dilated or arrhythmogenic cardiomyopathy?

**What is the main finding?**

Our analysis showed that participants who were heavy drinkers during young adulthood were on average 7.9 years younger at age of dilated or arrhythmogenic cardiomyopathy diagnosis.

Patients diagnosed with genetic DCM/ACM were recruited from the Cardiovascular Genetics Clinic at Brigham and Women’s Hospital from January 20, 2023, to October 6, 2025. Participants were defined as having genetic DCM/ACM if they had diagnostic disease features (left ventricular diastolic dysfunction, dilated ventricular remodeling, and/or fibrofatty replacement of cardiac myocytes) and carried a pathogenic or likely pathogenic variant in an established cardiomyopathy gene. Individuals with arrhythmogenic right ventricular cardiomyopathy (ARVC) were included in analysis, despite the differing natural history of ARVC compared to other ACMs, as the analysis does not consider disease progression beyond the initial date of diagnosis. Pathogenic or likely pathogenic variants in *DSP, BAG3, TTN, LMNA, DSG2, PKP2, FLNC, DES, MYH7, PLN, RBM20, SCN5A, TNNT2, ALMS1,* or *TMEM43* were included.

This study was approved by and received proper ethical oversight from the Mass General Brigham Institutional Review Board. Informed consent was obtained from all participants. Participants were asked to fill out a retrospective survey about alcohol use over their lifetime during their in-person clinical visit.^2^^,^^3^ Questions were framed in both multiple choice and numeric fill-in-the-blank formats. For each decade of their life, participants self-reported the maximum number of drinks they had within a 24-hour period.

Responses pertaining to the 21 to 30 age range were analyzed. Clinical information, including age of cardiomyopathy diagnosis and genetic variant, was obtained from a single center database. Survey respondents diagnosed with DCM/ACM before age 21 were excluded. Age of diagnosis was used as a proxy for disease onset. As defined by the National Institute on Alcohol Abuse and Alcoholism, participants were classified as “heavy drinkers” based on the maximum number of drinks consumed in a 24-hour period (≥4 drinks for women, ≥5 drinks for men). Heavy drinking and age of diagnosis were compared with a Welch 2 sample t-test.

Of the 307 participants who consented to taking the survey, a total of 284 participants completed the survey questions pertaining to alcohol use between ages 21 to 30 (response rate = 92.5%). After excluding respondents without confirmed DCM/ACM diagnoses (n = 48), no associated disease genes (n = 71), and diagnosis ages below age 21 (n = 31), 134 participants (age 49.8 ± 14.6 years; 60.4% females) including 9 participants with ARVC (6.7%) were analyzed (Table 1). Over half (n = 72; 53.7%) of the participants were classified as heavy drinkers during the ages of 21 to 30, whereas 62 (46.3%) participants were classified as nonheavy drinkers. Heavy drinkers were more likely to be White (97.2% vs 92.9%; *P* = 0.016). The 3 most common disease genes were *LMNA* (24.6%), *TTN* (21.6%), and *DSP* (19.4%) and did not differ significantly in heavy vs nonheavy drinkers.

**Table 1:** Demographic, Genetic, Survey, and Age of Diagnosis Data for Heavy and Nonheavy Drinkers

	Total	Heavy Drinkers^a^	Nonheavy Drinkers	*P* Value
n, %	134	72 (53.7%)	62 (46.3%)	0.388^b^
Female	81 (60.4%)	41 (56.9%)	40 (64.5%)	0.371^c^
Race				
White	127 (94.8%)	70 (97.2%)	57 (91.9%)	0.018^d^^,^^e^
Black	3 (2.24%)	0 (0.0%)	3 (4.8%)	
Unknown	2 (1.5%)	2 (2.8%)	0 (0.0%)	
Asian	2 (1.5%)	0 (0.0%)	2 (3.2%)	
Associated disease gene				
*LMNA*	33 (24.6%)	20 (27.8%)	13 (21.0%)	0.654^d^
*TTN*	29 (21.6%)	13 (18.1%)	16 (25.8%)	
*DSP*	26 (19.4%)	13 (18.1%)	13 (21.0%)	
*PKP2*	7 (5.2%)	5 (6.9%)	2 (3.2%)	
Other	39 (29.1%)	21 (29.2%)	18 (29.0%)	
Clinical diagnosis				
DCM/AC	125 (93.3%)	65 (90.3%)	60 (96.8%)	0.176^d^
ARVC	9 (6.7%)	7 (9.7%)	2 (3.2%)	
Reason for clinical diagnosis				
Event/symptoms	77 (57.5%)	41 (56.9%)	36 (58.1%)	0.421^d^
Cascade testing	44 (32.8%)	26 (36.1%)	18 (29.0%)	
Incidental finding	11 (8.2%)	5 (6.9%)	6 (9.7%)	
Unknown	2 (1.5%)	0 (0.0%)	2 (3.2%)	
Average age at diagnosis (y)	42.3 ± 13.0	38.6 ± 11.6	46.5 ± 13.3	<0.001^e^^,^^f^
Average age at diagnosis for selected disease genes (y)				
LMNA	39.1 ± 11.7	36.8 ± 10.5	42.6 ± 13.1	0.200^f^
TTN	46.6 ± 12.9	41.3 ± 13.5	50.8 ± 11.1	0.052^f^
DSP	45.1 ± 16.1	39.0 ± 14.6	51.2 ± 15.8	0.053^f^
PKP2	46.2 ± 13.0	42.3 ± 8.9	55.9 ± 20.8	0.279^g^

ACM = arrhythmogenic cardiomyopathy; ARVC = arrhythmogenic right ventricular cardiomyopathy; DCM = dilated cardiomyopathy.

a Heavy drinking definition: 4 or more drinks in 24 hours for women, 5 or more drinks in 24 hours for men.

b Chi-square test for given probabilities was used.

c Pearson chi-square test was used.

d Fisher exact test for count data was used to account for small sample sizes.

e *P* values are rounded to 2 decimal places for display; values near 0.05 should be interpreted using full precision. Statistical significance (signified by ∗) was defined as *P* < 0.05.

f A Welch 2 sample t-test was used to address unequal variances and account for non-normality with large sample sizes.

g An approximate two-sample Fisher-Pitman permutation test was used to account for small sample sizes.

Most participants were diagnosed with DCM/ACM after presenting with symptoms or a medical event (57.5%). Table 1 further delineates demographic and clinical data for the participant population.

Compared with heavy drinkers, participants who were nonheavy drinkers during young adulthood were 7.9 years (95% CI: 3.6-12.2 years) older at age of diagnosis (46.5 ± 13.3 years; 95% CI: 43.2-49.9 years vs 38.6 ± 11.6; 95% CI: 35.6-41.3 years; *P* < 0.001). When stratifying heavy drinking and non-heavy drinking groups by genes of interest, heavy drinkers were diagnosed at younger ages, although these differences did not reach statistical significance. A sensitivity analysis was conducted in the 77 participants who were diagnosed based on the presence of an event or symptoms (Table 1). There were 41 heavy drinkers (53.3%) and 36 nonheavy drinkers (46.7%) in this subgroup. Comparable with the overall findings, the median age at diagnosis was lower in the symptomatic heavy vs nonheavy drinking group (37.5 years (IQR: 29.3-46.2) vs 45.1 years (IQR: 39.3-55.7); *P* = 0.016).

This study represents a pilot analysis of the association between heavy drinking during young adulthood on age of diagnosis with DCM/ACM in genetically predisposed individuals. Heavy alcohol use was very common in this cohort and associated with nearly a decade younger age at diagnosis compared to those who did not report heavy drinking. This corroborates the current clinical understanding that alcohol can contribute to disease progression in at-risk variant carriers. Our findings validate and extend those reported by Ware et al^4^ which identified *TTN* truncating variants as a risk factor for the development of cardiomyopathy.

A larger sample size is needed to ascertain the potential gene-specific impact of early-life heavy drinking on age of DCM/ACM onset. The results of our study differ from an analysis from the DCM Precision Medicine cohort in which cumulative alcohol exposure was not found to have a significant effect on rates of DCM development in individuals with a family history.^5^ The adverse effect of alcohol identified in our study may be explained by restricting our analysis to genetically at-risk individuals and examining how heavy drinking, rather than overall alcohol exposure, affected the age of DCM diagnosis.

This study has several limitations. The association identified in this study should be interpreted in the context of survivorship, recall, and self-reporting biases. Moreover, the generalizability of the study is limited due to the status of the site as a tertiary care center and the relative lack of diversity in the study sample. As an exploratory study, we focused our analysis on heavy drinking rather than total alcohol consumption over the examined decade. This choice, influenced by the methodological constraints on calculating total alcohol consumption and the limitations of retrospective self-reporting, may result in nondifferential misclassification and downplay the cardiac burden of chronic alcohol use during young adulthood and throughout the lifespan. In addition, the study is limited by our use of diagnosis age as a proxy for disease onset, which may conflate age of clinical attention with biological onset of disease.

Despite these limitations, this underexplored association between heavy drinking and age of DCM/ACM diagnosis provides a foundation for future studies examining the role of alcohol exposure in disease expression among genetically predisposed individuals. Although this study focused on alcohol use, the observed association could have broader implications for other early-life toxin exposures, particularly drug use, which have been associated with cardiomyopathy development. Further research, including a prospective analysis of lifetime alcohol consumption with adjustment for confounding variables and other cardiac risk factors, is needed to strengthen clinical guidelines regarding alcohol use for young adults with a genetic predisposition to cardiomyopathy, especially given the high prevalence of alcohol use in this population.

## Funding support and author disclosures

Dr Bart has received research funding from Pfizer; speaking fees from Pfizer; and advisory board fees from Bridge Bio, Novo Nordisk, and Bristol Myers Squibb. Dr Ho has received unrestricted research support and/or consulting fees from Bristol Myers Squibb, Cytokinetics, Lexicon, Alexion, Astra Zeneca, Novo Nordisk, and Sanofi. Dr Lakdawala has received unrestricted research funding from the O'Hare, Corkin and Steggall family research foundations, BMS, and Pfizer; is partially supported by the British Heart Foundation’s Big Beat Challenge award to CureHeart (BBC/F/21/220106); and has received modest consulting incomes from BMS, Pfizer, Bayer, Kardigan, Cytokinetics, Bridge Bio, Flux and Lexeo. All other authors have reported that they have no relationships relevant to the contents of this paper to disclose.
